# Effects of exercise-based cardiac rehabilitation in patients after percutaneous coronary intervention: A meta-analysis of randomized controlled trials

**DOI:** 10.1038/srep44789

**Published:** 2017-03-17

**Authors:** Xinyu Yang, Yanda Li, Xiaomeng Ren, Xingjiang Xiong, Lijun Wu, Jie Li, Jie Wang, Yonghong Gao, Hongcai Shang, Yanwei Xing

**Affiliations:** 1 Guang’anmen Hospital, Chinese Academy of Chinese Medical Sciences, Beijing 100053, China; 2 The Key Laboratory of Chinese Internal Medicine of the Ministry of Education, Dongzhimen Hospital Affiliated to Beijing University of Chinese Medicine, Beijing 100700, China; 3 Beijing University of Chinese Medicine, Beijing 100029, China

## Abstract

In this study, we assessed the effect of rehabilitation exercise after percutaneous coronary intervention (PCI) in patients with coronary heart disease (CHD). We performed a meta-analysis to determine the effects of exercise in patients after PCI. The Cochrane Central Register of Controlled Trials (CENTRAL), PubMed, the Embase database, China National Knowledge Internet (CNKI), China Biology Medicine (CBM), and the Wanfang Database were searched for randomized controlled trials (RCTs). The key words used for the searches were PCI, exercise, walking, jogging, Tai Chi, and yoga. Six studies with 682 patients met our inclusion criteria; we chose the primary endpoint events of cardiac death, recurrence of myocardial infarction (MI), repeated PCI, coronary artery bypass grafting (CABG), and restenosis, and the secondary endpoint measures included recurrent angina, treadmill exercise (total exercise time, ST-segment decline, angina, and maximum exercise tolerance). The results showed that exercise was not clearly associated with reductions in cardiac death, recurrence of MI, repeated PCI, CABG, or restenosis. However, the exercise group exhibited greater improvements in recurrent angina, total exercise time, ST-segment decline, angina, and maximum exercise tolerance than did the control group. Future studies need to expand the sample size and improve the quality of reporting of RCTs.

Coronary heart disease (CHD), also known as coronary artery disease (CAD), is a common cause of heart disease-related death globally that led to 7.4 million deaths in 2013, which was one-third of all deaths (WHO 2014)^1^. When treating CHD, interventions that are designed to decrease the mortality rate may reduce the probability of dying from CHD, such as from a heart attack^2^. Patients with symptoms of angina despite optimal medical therapy require mechanical revascularization with either CABG or PCI. PCI has been shown to effectively reduce mortality and morbidity in patients with CAD^3^. This method refers to post-cardiac catheterization technology, such as dredge stenosis or occlusion of the coronary artery lumen to improve myocardial perfusion treatment^4^,^5^. PCI is an effective treatment strategy for coronary artery stenosis^6^. This procedure is relatively safe, and the benefit of PCI in patients is primarily symptom reduction^4^. However, the PCI operation may cause coronary spasm, endothelial cell injury, and even restenosis or thrombus^6^. The fragments derived from thrombi or atherosclerotic plaques may induce coronary artery embolization, which could result in myocardial injury or myocardial ischemia^5^,^6^,^7^,^8^. It is necessary to improve the rehabilitation efforts for cardiovascular system disease patients because such efforts can prolong survival time and improve quality of life^9^,^10^.

Cardiac rehabilitation is a fundamentally comprehensive intervention composed of exercise training, risk factor education, psychological support, life-style behavior changes, and multiple approaches to handling common CHD risk factors^11^,^12^,^13^. Activity is the best medicine^14^. Over the past several decades, numerous epidemiological studies have shown that exercise has beneficial effects on human health, such as reducing the risk of dementia^15^, providing protection against metabolic disorders^16^, and improving quality of life^17^. Exercise has become popular after PCI, especially during PCI operative rehabilitation, to help alleviate the patients’ symptoms; exercise is often combined with routine therapy. The American Heart Association (AHA) guidelines^18^ recommend training as a core element of exercise-based cardiac rehabilitation^19^. Exercise-based cardiac rehabilitation is aimed at improving the health and outcomes of people after PCI. Exercise includes walking, jogging, aerobic exercise, bicycling, stretching and calisthenics, pedaling, physical exercise, climbing up and down stairs, Tai Chi, yoga, qigong, boating, climbing, swimming, and tennis. Some studies have demonstrated that exercise is good for rehabilitation and recurrent symptoms after PCI. Few advantages of cardiac rehabilitation in this patient population have been documented. Reports of advantages that translate to measurable clinical outcomes following PCI are few. This systematic review aims to answer the following questions: (1) does exercise affect the primary endpoint events of cardiac death, recurrence of MI, repeated PCI, CABG, and restenosis after PCI? (2) Is exercise an effective method for improving secondary endpoint measures, such as quality of life, physical function, and symptoms, after PCI?

## Methods

### Data searches and sources

In this study, the Cochrane Central Register of Controlled Trials (CENTRAL), PubMed, the Embase database, CBM, China National Knowledge Internet (CNKI), and the Wanfang Database were searched for original studies; specific keywords and a search strategy were used. The key words used for the searches were “percutaneous coronary intervention (PCI)”, “exercise”, “walking”, “jogging”, “Tai Chi”, and “yoga”. Studies in the CENTRAL, PubMed, CBM, CNKI, and Wanfang databases that were written in Chinese or English and were related to the study were retrieved. Then, a careful analysis of literature titles, abstracts, keywords, and subject terms was performed to further determine the keywords for document retrieval. If the abstract was relevant to this study, we read the full text. The references in the studies were also analyzed to identify studies that may have been missed in the original searches. Searches of studies from the inception of the database to January 2016 were conducted in the search session, and prospective randomized controlled trials were chosen. The search was llimited to clinical trials with human participants.

### Study selection

The inclusion criteria for document selection were as follows: identified studies had to be RCTs of exercise and pharmacological therapy versus usual care and pharmacological therapy and had to have a follow-up of at least 2 months, regardless of blinding. In the meta-analysis, male and female participants of any age who had a history of PCI and were treated in a hospital were included. The study had to include data for both an exercise group and a control group. Participants who had modifiable cardiovascular risk factors or severe complications were excluded from the meta-analysis.

### Data extraction and management

Two authors (Yanda Li and Xiaomeng Ren) extracted the data and reviewed the eligibility and methodological quality of each included study. Any disagreements were discussed, and if the discussion did not yield a final decision, disagreements were resolved by the 3rd author (Xinyu Yang). First, the information extracted from each document included the first author’s name, the publication year, the sample size of the trial, the type of participants, clinical features, ages of participants, the style of exercise after PCI, the study duration, and the frequency of the exercise. Furthermore, we extracted data according to primary outcomes, which included cardiac death, recurrence of MI, repeated PCI, CABG, and restenosis, and the secondary end measures, which included recurrent angina, treadmill exercise (total exercise time, ST-segment decline, angina, and maximum exercise tolerance) during the follow-up period.

### Quality assessment

The risk of bias in the included literature was assessed using the criteria recommended by the Cochrane Collaboration. Study quality was evaluated according to the quality assessment criteria for RCTs in included studies using the Cochrane Collaboration’s recommended tool, including random sequence generation, blinding of participants and personnel, allocation concealment, incomplete outcome data, blinding of outcome assessment, selective reporting, and other biases.

### Statistical analysis

Dichotomous outcomes were analyzed via odds ratios (ORs) with 95% confidence intervals (CIs), and continuous variables were analyzed via standardized mean differences (SMDs)^20^ with 95% CIs. Analyses of the pooled data for outcome indicators, such as cardiac death, recurrence of MI, repeated PCI, CABG, restenosis, and recurrent angina were conducted using the DerSimonian and Laird random effects (RE) model. The analyses were then repeated with a fixed effects (FE) Mantel-Haenszel model to check for any possible differences. Overall heterogeneity was quantified with the Q-statistic, and its significance was tested with the Chi-square test, with *p* < 0.10 used to indicate significance^21^. The proportion of true variance in the estimated effects between the included studies, as opposed to sampling error within the studies, was calculated by the *I*^*2*^ statistic and considered high for an *I*^*2*^ *>* 75%^21^. We extracted the continuous outcomes, such as treadmill exercise, total exercise time, ST-segment decline, angina, and maximum exercise tolerance. The mean difference (MD) can be used as a summary statistic in meta-analyses when the outcome measurements in all studies are performed using the same scale. The standardized mean difference was used if the same outcome was measured in a variety of ways. Eleven authors provided data for the outcomes of interest, but one study did not report the SDs of the means. We thus used t-values to calculate the SDs^22^. The pooled analyses were conducted using the Review Manager 5.3 software (http://ims.cochrane.org/revman/download). Because this was a meta-analysis, ethical approval was not required.

## Results

### Selection and inclusion of studies

**Figure 1:**
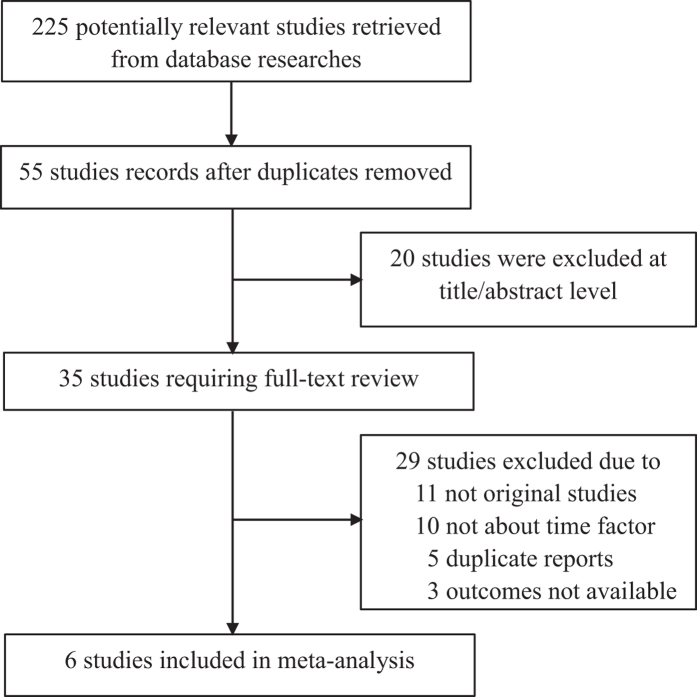
Flow chart of the search strategy used to identify trials for inclusion in the meta-analysis. RCT, randomized controlled trial.

A total of 157 studies were identified in the database with the aforementioned keywords, and 68 studies were identified from other sources. After 156 duplicates were excluded, some additional studies were excluded based on the study theme and abstract because they were not relevant to our topic. A total of 6 full-text studies were assessed for eligibility. Additional studies were excluded because they were meta-analyses or case studies, data for the exercise group were not available, the outcomes of interest were not reported, or dichotomous data that were very important for our statistical analysis were not provided. The flow diagram for the selection of studies is shown in Fig. 1.

### Characteristics of included studies

A total of 6 RCTs and observational studies were included in this meta-analysis, with a total of 682 patients. Among those patients, 341 were in exercise groups, and 341 were in control groups. Data on the characteristics of the studies and patients, the total number of patients in the different groups (including the control and exercise groups), essential drugs and care, intervention strategies, follow-up period, and clinical outcomes were shown in Table 1.

### Risk of bias in the included studies

**Figure 2:**
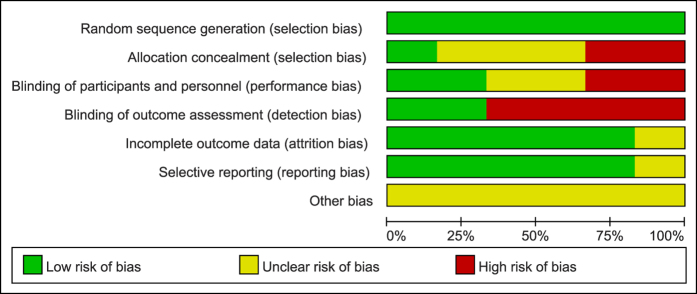
Quality assessment of the included studies in this review: Risk of bias graph.

**Figure 3:**
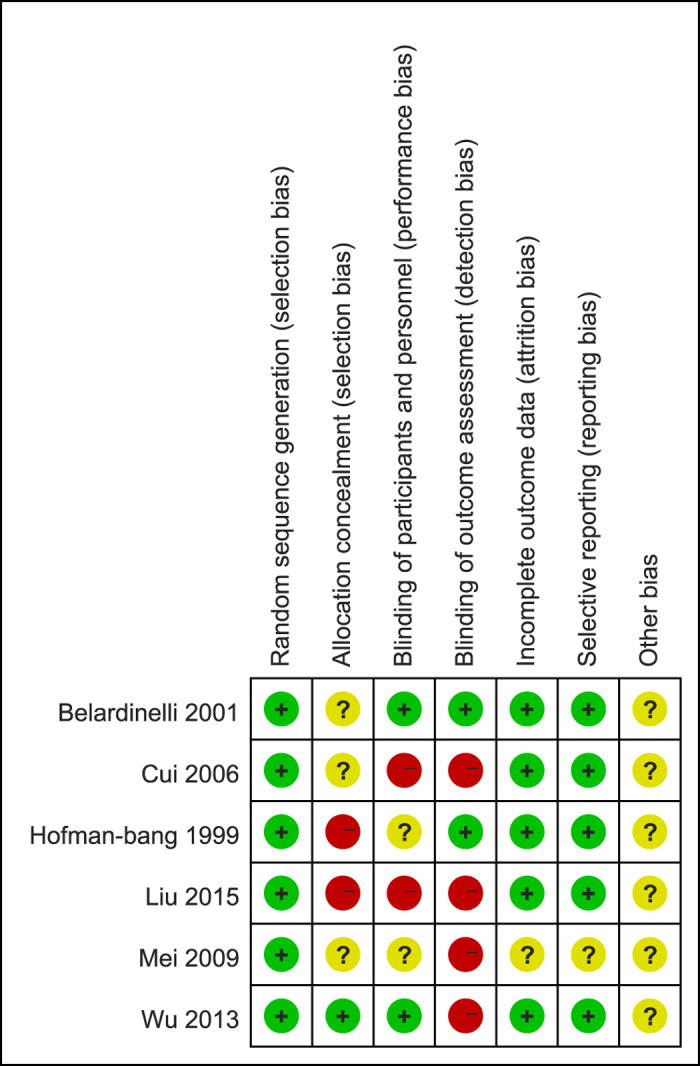
Quality assessment of included studies in this review: Risk of bias summary.

Judgments regarding every risk-of-bias item for all of the trials are shown in Figs 2 and 3. All of the studies described the specific methods used for random sequence generation. One document^23^ presented the allocation concealment in detail, two trials^24^,^25^ did not apply allocation concealment, and the other studies were unclear about this process. Blinded methods were used in two trials^23^,^26^, one^26^ of which used a blinded approach for investigators, participants, and outcome assessors, and one trial^23^ was only blinded for investigators and participants. One study^24^ was not clear regarding the blinding approach, and four studies^25^,^27^,^28^ were ranked as having a high risk of bias. None of the included documents had incomplete outcome data or selective reporting. Other sources of bias were not described in the trials (Figs 2 and 3).

### Primary endpoint events

#### Cardiac death

**Figure 4:**
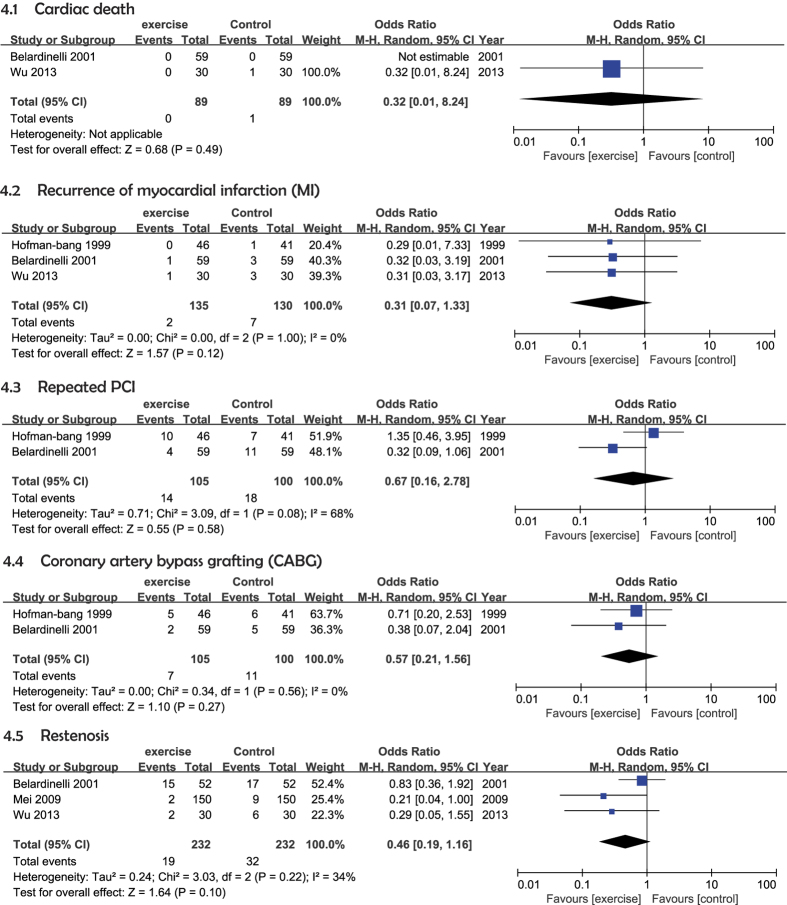
Forest plot of the meta-analysis of an exercise group and a control group with respect to the primary endpoint events. (4.1) Cardiac death; (4.2) recurrence of MI; (4.3) repeated PCI; (4.4) CABG; (4.5) restenosis.

Two studies investigated cardiac death during the follow-up period, and included a total of 205 participants (105 in the exercise group and 100 in the control group) Fig. 4^23^,^26^. With the use of a random-effects model, we found that exercise was not associated with significantly improved cardiac death rates (OR = 0.32 [95% CI (0.01–8.24)], *P* = 0.49) (Fig. 4.1).

#### Recurrence of myocardial infarction (MI)

Three included studies discussed the recurrence of MI during the follow-up period and included a total of 265 participants (135 in the exercise group and 130 in the control group)^23^,^24^,^26^. The random-effects model showed that exercise was not associated with a significantly improved recurrence of MI (OR = 0.31 [95% CI (0.07–1.33)], *P* = 0.12). The threshold for heterogeneity was *P* = 1.00 (*I*^*2*^ = 0%) (Fig. 4.2).

#### Repeated PCI

Two of the included studies investigated repeated PCI during the follow-up period and included a total of 205 participants (105 in the exercise group and 100 in the control group)^24^,^26^. The random-effects model showed that exercise was not associated with significantly improved repeated PCI rates (OR = 0.67 [95% CI (0.16–2.78)], *P* = 0.58). The heterogeneity was *P* = 0.08 (*I*^2^ = 68%) (Fig. 4.3).

#### Coronary artery bypass grafting (CABG)

Two trials showed CABG rates during a follow-up period with a total of 205 participants (105 in the exercise group and 100 in the control group)^24^,^26^. We used a random-effects model and found that exercise was not associated with significantly improved CABG rates (OR = 0.57 [95% CI (0.21–1.56)], *P* = 0.27). The heterogeneity was *P* = 0.56 (*I*^2^ = 0%) (Fig. 4.4).

#### Restenosis

**Figure 5:**
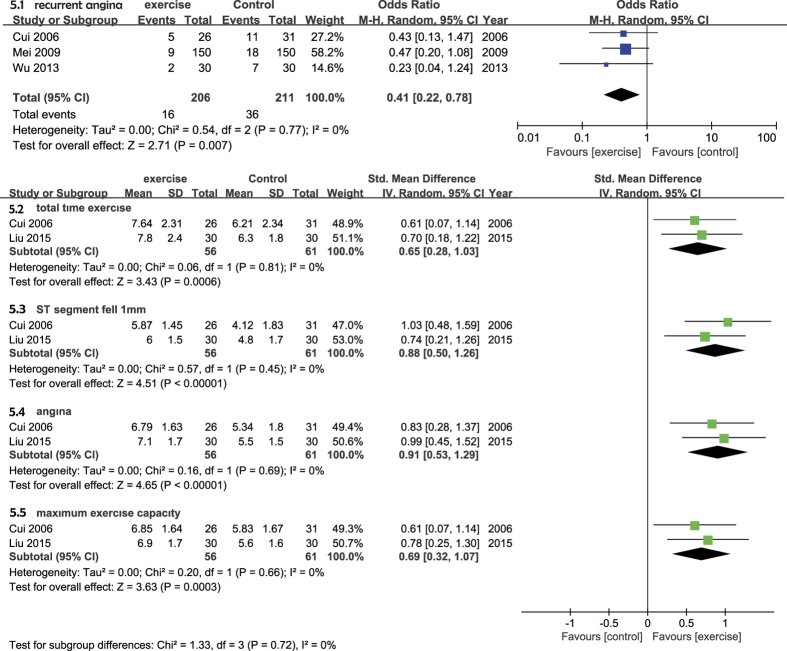
Forest plot of the meta-analysis of two studies with an exercise group and a control group with respect to the secondary endpoint measures. (5.1) Recurrent angina and treadmill exercise: (5.2) total time exercise; (5.3) ST-segment decline; (5.4) angina; (5.5) maximum exercise tolerance.

Three of the included studies assessed restenosis during the follow-up period, and included a total of 478 participants (239 in the exercise group and 239 in the control group)Fig. 5^23^,^26^,^27^. The random-effects model showed that exercise was not associated with significantly improved restenosis rates (OR = 0.46 [95% CI (0.19–1.16)], *p* = 0.10). The heterogeneity was *P* = 0.22 (*I*^2^ = 34%) (Fig. 4.5).

### Secondary endpoint measures

#### Recurrent angina

Three trials assessed recurrent angina during the follow-up period and included a total of 417 participants (206 in the exercise group and 211 in the control group) Fig. 5^23^,^27^,^28^. We used a random-effects model and determined that exercise was associated with significantly improved recurrent angina rates (OR = 0.41 [95% CI (0.22–0.78)], *P* = 0.007). The heterogeneity was *P* = 0.77 (*I*^2^ = 0%) (Fig. 5.1).

#### Treadmill exercise

Two of the included studies assessed treadmill exercise during the follow-up period and included total of 117 participants (56 in the exercise group and 61 in the control group)^25^,^28^. In the meta-analysis, we chose to assess the following four variables: total exercise time, ST-segment decline, angina, and maximum exercise tolerance. The pooled results indicated that the individuals in the exercise group were significantly more likely to recover than those in the control group. A random-effects model was chosen due to a lack of potential heterogeneity between trials (*I*^*2*^* = *0%, *P* = 0.94). Exercise was associated with a significantly improved total exercise time (SMD = 0.65 [95% CI (0.28–1.03)], *P* = 0.0006) (Fig. 5.2), ST-segment elevation (SMD = 0.88 [95% CI (0.50–1.26)], *P* < 0.00001) (Fig. 5.3), angina (SMD = 0.91 [95% CI (0.53–1.29)], *P* < 0.00001) (Fig. 5.4), and maximum exercise tolerance (SMD = 0.69 [95% CI (0.32–1.07)], *P* = 0.0003). The threshold heterogeneity revealed the following: *P* = 0.81 and *I*^*2*^ = 0%, *P* = 0.45 and *I*^*2*^ = 0%, *P* = 0.69 and *I*^*2*^ = 0%, *P* = 0.66 and *I*^*2*^ = 0% for total exercise time, ST-segment elevation, angina, and maximum exercise tolerance, respectively (Fig. 5.5).

#### Sensitivity analysis

Heterogeneity across studies was tested with the *I*^2^ statistic developed by Higgins^29^, which provides a better measure of the consistency between trials in a meta-analysis. Studies with an *I*^2^ statistic of 25–50% were considered to have low heterogeneity, those with an *I*^2^ statistic of 50–75% were considered to have moderate heterogeneity, and those with an *I*^2^ statistic of >75% had a high degree of heterogeneity. A high degree of heterogeneity may have been due to a small study number, protocol differences, small sample sizes, or varying backgrounds of the participants.

## Discussion

To our knowledge, this is the first meta-analysis about the effects of exercise-based cardiac rehabilitation for CHD patients who have undergone PCI. Our review incorporated 6 studies and 682 participants who presented with coronary heart disease after PCI. The purpose of our meta-analysis was to determine whether exercise could reduce endpoint events or improve the physiological state of PCI patients. In this analysis of studies, we found that (1) exercise was not clearly associated with reductions in cardiac death, the recurrence of MI, repeated PCI, CABG, or restenosis and (2) exercise could improve recurrent angina, total exercise time, ST-segment decline, angina, and maximum exercise tolerance after PCI.

Cardiac death, the recurrence of MI, repeated PCI, CABG, and restenosis are important risk factors for post-operative CHD. It was expected that these risk factors would decrease as a result of exercise post-operatively, but the results were not statistically significant in our study. In one of the selected studies, Belardinelli *et al*. showed that the exercise groups had fewer events and a lower hospital readmission rate than controls, despite an unchanged restenosis rate^26^. Hofman-Bang *et al*. showed that the achieved changes were maintained after 2 years of follow-up and were accompanied by a significant improvement in exercise capacity^24^. Although the result was not significant, the exercise group tended to exhibit a reduction in endpoint events compared with the control group. Exercise may induce beneficial effects such as collateral formation and improved endothelial function, which reduce ischemia^30^. Therefore, exercise could still have been responsible for this post-operative rehabilitation.

Recurrent angina was a secondary endpoint measure. It is associated with an exercise improvement in cardiac rehabilitation after PCI. The PCI operation may cause coronary spasm or endothelial cell injury, lead to coronary problems, and cause acute myocardial ischemia and hypoxia. In this study, the incidence of recurrent angina after PCI was lower in the exercise group than in the control group. Previous reports have suggested the necessity of exercise training, which improves exercise capacity and increases the supply of oxygen to cardiac muscles (measured by peak myocardial oxygen consumption) in patients with coronary artery disease^31^. As a form of post-PCI rehabilitation, exercise could activate the natural self-healing ability and evoke the balanced release of endogenous neurohormones and various natural health recovery mechanisms to improve collateral circulation in the heart and increase activity tolerance^32^; exercise may also increase the coronary blood flow reserve capacity and cardiovascular work efficiency to accelerate physical recovery and avoid and reduce the occurrence of recurrent angina.

In this meta-analysis, the four indicators that were selected from treadmill exercise were total exercise time, ST-segment decline, angina, and maximum exercise tolerance. The results from our study revealed an improvement in the exercise group compared with the control group after PCI. According to the data in this study, treadmill exercise was associated with higher exercise capacity and maximal oxygen uptake after PCI. Some studies showed that exercise can enhanced physical capacity and lead to improved daily physical activity^33^,^34^, which would favorably influence an individual’s lifestyle^35^. Rehabilitation exercise may increase blood flow to the heart and increase the myocardial contraction force, improve the heart pumping strength, help reduce and maintain a healthy weight, and effectively control blood pressure, blood sugar, and blood fat. Rehabilitation exercise can also help reduce stress, enhance vitality, and relieve pain^9^,^10^.

This study showed that the exercise group had improvements in recurrent angina, total exercise time, ST-segment decline, angina, and maximum exercise tolerance compared with the control group following PCI. However, recurrent angina, total exercise time, ST-segment decline, angina, and maximum exercise tolerance in the group subjected to exercise do not appear to reflect a better outcome of clinically relevant aspects such as cardiac death, recurrence of MI, repeated PCI, CABG, and restenosis. Although the result was not significant, the exercise group tended to exhibit a reduction in endpoint events compared with the control group. We also found that exercise could be beneficial for refractory angina before and after PCI^23^,^27^,^28^; angina attacks were decreased, exercise tolerance was increased and quality of life was improved^9^,^10^. Therefore, we propose the following explanations: (1) Due to the limited quantity of studies, the small sample size and the short follow-up time, it is difficult to determine whether cardiac rehabilitation can reduce the primary end point events. Future studies need to expand the sample size and improve the quality of reporting of RCTs. (2) Although rehabilitation exercise can help reduce stress, enhance vitality, and relieve pain^9^,^10^, cardiac rehabilitation may not be able to block the development of coronary artery atherosclerosis, inhibit the occurrence of vascular inflammation, or make plaque more stable; ultimately, cardiac rehabilitation cannot effectively reduce the primary endpoint events. Atherosclerosis is the major cause of myocardial infarction and stroke and the leading cause of death worldwide^36^. Atherosclerosis is a chronic inflammatory disease of the arteries – inflammation is present and is mediated by different chemokines/cytokines at all stages – from leukocyte recruitment by adhesion molecules in plaque formation to collagen cap digestion by metalloproteinases (MMPs), which contributes to plaque instability. More research is needed to determine whether cardiac rehabilitation can prevent the progression of atherosclerosis.

There were some limitations in this meta-analysis. Primarily, the poor level of reporting in the included RCTs made it difficult to evaluate study quality and to judge the risk of bias. Second, two of the six studies that were included in the meta-analysis did not include a sufficiently long follow-up time. Finally, heterogeneity existed in our meta-analysis due to the limited quantity of studies, the small sample size, different protocols, and different participant backgrounds. Thus, high-quality studies are required to identify the effect of exercise as a cardiac rehabilitation modality after PCI for a longer follow-up time.

In conclusion, this study showed that exercise was not clearly associated with reductions in cardiac death, the recurrence of MI, repeated PCI, CABG, or restenosis. However, the exercise group exhibited improvements in recurrent angina, total exercise time, ST-segment decline, angina, and maximum exercise tolerance compared with the control group following PCI. Future studies also need to expand the sample size and improve the quality of reporting.

## Additional Information

**How to cite this article:** Yang, X. *et al*. Effects of exercise-based cardiac rehabilitation in patients after percutaneous coronary intervention: a meta-analysis of randomized controlled trials. *Sci. Rep.* **7**, 44789; doi: 10.1038/srep44789 (2017).

**Publisher's note:** Springer Nature remains neutral with regard to jurisdictional claims in published maps and institutional affiliations.

## Figures and Tables

**Table 1:** Characteristics of randomized controlled trials included in this meta-analysis.

Study	Participants (E/C)	Surgical procedure	Age (E/C)	Sex (F/M)	Exercise Group		Control group	Follow-up	Outcome measures
					Mode of exercise	Exercise program			
Belardinelli *et al*.^26^	59/59	PCI	E53.0 ± 11.0, C59.0 ± 10.0	49/50	Stretching and calisthenics, pedaling	Every time: 30 minutes Frequency: 3 times per week Total duration: 6 months	Pharmacological therapy	6 months	Cardiac death, recurrence of MI, repeated PCI, restenosis, CABG
Wu *et al*.^23^	30/30	PCI	E68.2 ± 12.04, C 68.3 ± 10.9	19/15	Walking, jogging, bicycling, tai chi	Every time: 20–40 minutes Frequency: 5–7 times per week Total duration: 6 months	Pharmacological therapy, Post-operative management	6 months	Cardiac death, recurrence of MI, restenosis, recurrent angina,
Hofman-Bang *et al*.^24^	46/41	PCI	E 53.0 ± 7.0, C 53.0 ± 7.0	37/36	Physical exercise	Duration and Frequency: not mentioned Total duration:12 months	Standard care	24 months	Recurrence of MI, repeated PCI, CABG,
Mei *et al*.^27^	150/150	PCI	E 64.0 ± 9.1, C 64.0 ± 9.1	Not mentioned	Walking	Every time: 10–20 minutes. Frequency: 2 times/day;14 times/week. Total duration: 6 months.	Usual care	6, 12, 38 months	Restenosis, recurrent angina
Cui *et al*.^28^	26/31	PCI	E 59.4 ± 5.9 C 58.3 ± 6.1	21/23	Walking, bicycling, boating, pedaling	Every time: 5–10 minutes. Frequency: 3–4times/day;6-12 times/week. Total duration: 3 months.	Pharmacological therapy	3 months	Total exercise time, ST-segment decline, angina, Maximum exercise tolerance, recurrent angina
Liu *et al*.^25^	30/30	PCI	E 65.2 ± 5.6, C 65.2 ± 5.6	Not mentioned	Walking, climbing up and down stairs	Every time: 30 minutes. Frequency:2 times/day;14 times/week. Total duration: 3 months.	Pharmacological therapy, Post-operative management	3 months	Total exercise time,ST-segment decline, angina, maximum exercise tolerance

PCI = percutaneous coronary intervention; E = exercise group; C = control group; F = female; M = male; MI = myocardial infarction; CABG = coronary artery bypass grafting.
